# Extracorporeal Shock Wave Therapy and the Handstand-Position Radiography for Proximal Humeral Epiphysiolysis in Elite Gymnasts: A Report of Two Cases

**DOI:** 10.7759/cureus.60394

**Published:** 2024-05-15

**Authors:** Toru Omodani, Norimasa Takahashi, Kenji Takahashi

**Affiliations:** 1 Orthopaedics, Tokyo Advanced Orthopaedics, Tokyo, JPN; 2 Sports Medicine and Joint Center, Funabashi Orthopaedic Hospital, Funabashi, JPN

**Keywords:** return to sports, gymnast, handstand-position radiography, extracorporeal shock wave therapy, proximal humeral epiphysiolysis

## Abstract

We report two cases of proximal humeral epiphysiolysis in elite gymnasts. Both patients presented with shoulder pain during weight-bearing movements. The patient in case 1, treated with extracorporeal shock wave therapy (ESWT), exhibited rapid bone repair and pain relief, allowing an early return to competition. In the case 2 patient, humeral shortening was identified. Handstand-position radiography revealed compensatory scapular movements, negating the need for surgical intervention. These findings highlight ESWT's potential in promoting bone repair and the utility of handstand-position radiography in assessing humeral length. Both methods provide innovative treatment approaches for proximal humeral epiphysiolysis in gymnasts.

## Introduction

Proximal humeral epiphysiolysis is one of the overuse injuries that occur in juvenile athletes [1]. In baseball, it is a common injury caused by pitching and is also referred to as Little Leaguer's shoulder [2]. It is believed to develop as a result of accumulated damage to the proximal epiphysis of the humerus due to sports activities [3]. Most athletes can return to competition after adequate rest, and it is rare that surgery is required [4]. To date, there have been no reports of proximal humeral epiphysiolysis occurring in gymnasts. We report two cases of proximal humeral epiphysiolysis in elite-level gymnasts treated with extracorporeal shock wave therapy (ESWT) and handstand-position radiography.

## Case presentation

Case 1

**Figure 1:**
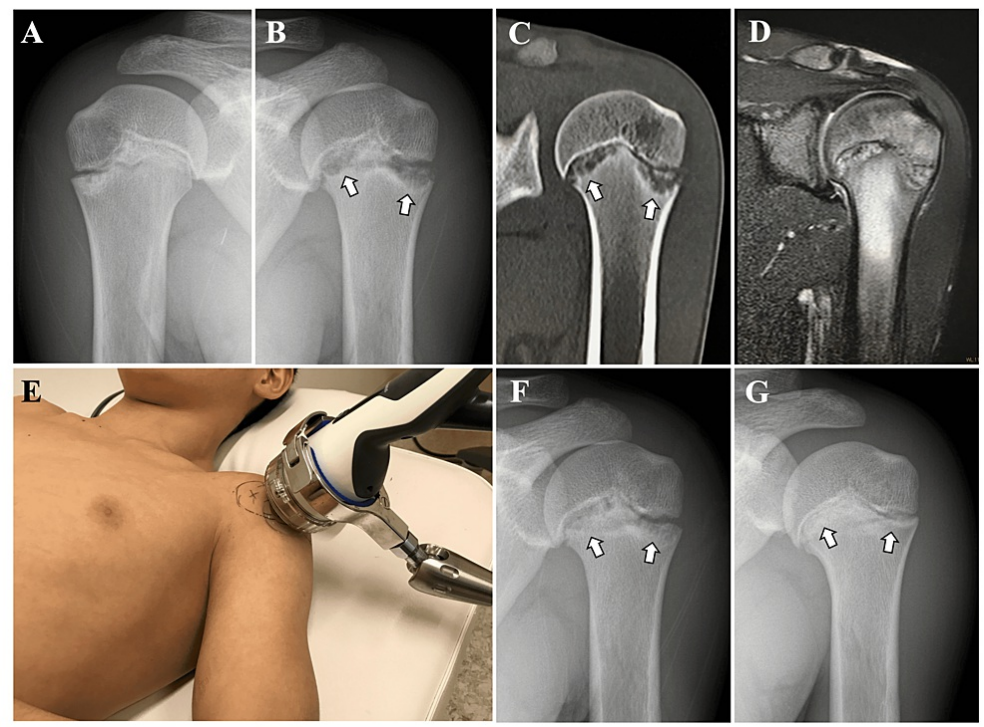
Case 1 A: Radiograph of the healthy side. B, C: Radiograph and computed tomography showed bone resorption on the distal side of the proximal humeral physis (arrows). D: T2-weighted fat-suppressed magnetic resonance imaging showed high signal changes centered around the epiphysis. E: Focused extracorporeal shock wave therapy (ESWT) was applied to the lateral side of the proximal humerus. F: Two months after initiating ESWT, vigorous bone repair was observed (arrows). G: The radiograph seven months after the start of ESWT showed good bone repair (arrows).

A 13-year-old male gymnast, intent on participating at the international competition level, presented to our clinic with ongoing left shoulder pain for three months during weight-bearing movements such as handstands and pommel horse skills. Tenderness was observed on the lateral side of the proximal end of the humerus. Radiograph and computed tomography showed bone resorption on the distal side of the proximal humeral physis compared to the healthy side (Figure 1 A-C). T2-weighted fat-suppressed magnetic resonance imaging showed high signal changes centered around the epiphysis (Figure 1D). Based on the physical and imaging findings, he was diagnosed with proximal humeral epiphysiolysis. While rest is generally recommended, the athlete had a strong desire to participate in the national team selection competition in three months. After discontinuing exercises that put weight on the painful limb, we decided on a treatment plan using extracorporeal shock wave therapy (ESWT) with the aim of promoting bone repair and alleviating pain.

The patient was positioned supine on the bed, and focused ESWT was applied to the lateral side of the proximal humerus (Figure 1E). Using the DUOLITH SD1 (STORZ MEDICAL AG, Switzerland), 2,500 shots were delivered at an energy of 0.20mJ/mm^2^, every month for a total of four sessions. He continued with training exercises that did not cause pain, excluding movements like pommel horse circles.

Two months after initiating ESWT, vigorous bone repair was observed, prompting the resumption of weight-bearing exercises on the affected limb (Figure 1F). One month after resuming training, the numerical pain rating scale improved from 10 pre-ESWT to 1. The performance level returned to pre-injury status. He competed in the targeted competition and was selected as a representative for international competitions. The radiograph, seven months after the start of ESWT, showed good bone repair (Figure 1G). Five years post-treatment, he continues his gymnastics at an elite level without any recurrences or sequelae.

Case 2

**Figure 2:**
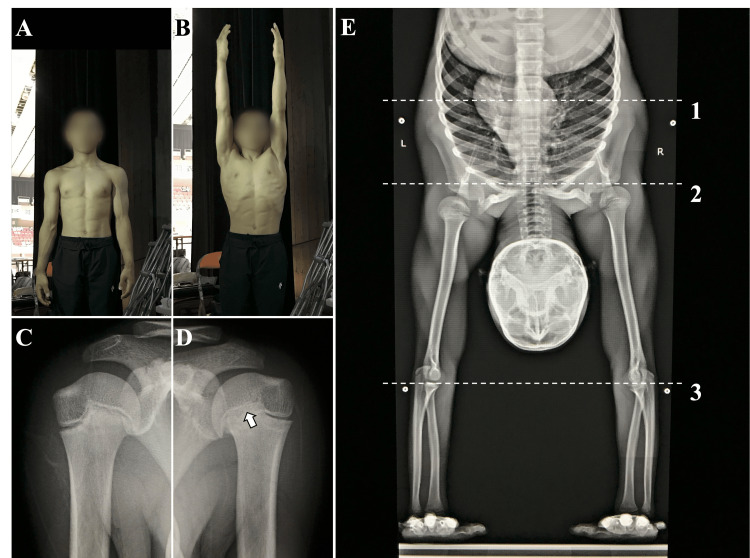
Case 2 A, B: When the arm was in a hanging position, the affected side was shorter in limb length. On the other hand, when the upper limb was raised, there seemed to be no difference in limb length on the left side. C: Radiograph of the healthy side. D: Radiograph revealed a closure of the medial portion of the proximal humeral physis compared to the healthy side  (arrows). E: A full limb radiograph in the handstand position. The humeral length was shortened, but the handstand was executed due to the compensatory movement of the scapula. Line 1: Height of the distal edge of the healthy side's scapula. Line 2: Height of the glenohumeral joint of the healthy side. Line 3: Height of the radioulnar joints on both sides.

A 15-year-old male gymnast, competing at the international competition level, presented to our clinic with ongoing left shoulder pain for six months during weight-bearing movements such as handstands and pommel horse skills. Tenderness was observed on the lateral side of the proximal end of the humerus. Additionally, when the arm was in a hanging position, the affected side was shorter in limb length (Figure 2A). On the other hand, when the upper limb was raised (Figure 2B), there seemed to be no difference in limb length on the left side. He did not perceive any difference in upper limb length during handstands. Radiograph revealed a closure of the medial portion of the proximal humeral physis compared to the healthy side (Figure 2 C,D). Based on the physical and imaging findings, he was diagnosed with premature closure of the physis and shortening of the humerus associated with proximal humeral epiphysiolysis.

A treatment strategy needed to be determined for the shortened humerus. To assess the impact of humeral shortening on gymnastic movements, a full-limb radiograph in the handstand-position was conducted (Figure 2E). The distance from the weight-bearing palm area to the glenohumeral joint was decreased on the affected side. On the other hand, increased abduction and elevation of the scapula were noted on the affected side. This suggested that unconscious compensatory movements of the scapula were occurring, making the athlete unaware of the humeral shortening during competition. Surgical procedures, such as humeral lengthening, were not chosen. Instead, a decision was made to carefully observe the progress while the athlete continued gymnastics. Rehabilitation by a physical therapist involved conservative treatments such as improving the range of motion of the glenohumeral and scapulothoracic joints, and strengthening the core. Five years after the onset, there is still a difference in limb length between the left and right, but there are no particular symptoms. He continues to compete in gymnastics at the international competition level.

## Discussion

Case 1 focused on treatment, while Case 2 focused on diagnosis.

In case 1, ESWT was administered with the aim of promoting bone repair and alleviating pain. ESWT is widely used in the treatment of musculoskeletal disorders. It is considered effective for fractures, tendinopathy, muscle injuries, osteoarthritis, and muscle spasticity [5-7]. To date, there have been no reports of ESWT being administered for proximal humeral epiphysiolysis. Shock waves on bones promotes the production of substances such as transforming growth factor-beta 1, endothelial nitric oxide synthase, and vascular endothelial growth factor, which in turn stimulate angiogenesis and bone formation [8-11]. Furthermore, it is known that shock waves can selectively destroy unmyelinated nerves and inhibit the conduction of neurotransmitters, making it effective in pain relief [12,13]. Additionally, there have been reports that multiple applications of shock waves can suppress the regeneration of destroyed nerves [14]. Considering these mechanisms, there have been reports that ESWT is effective for epiphysiolysis in juvenile athletes [15, 16]. In baseball, it has been reported that the average time to fully recover from proximal humeral epiphysiolysis is 2.8 months including a period of prohibited pitching [17]. After returning, 25% of players reportedly experience a recurrence of pain within an average of 3.5 months. A study indicates that complete bone repair takes about 4.7 months radiographically, and it is recommended not to allow athletes to return to competition until this recovery is achieved [18]. For case 1, the patient did not take a complete break from training and resumed full-fledged competitive practice within two months. This recovery was quicker than what has been previously reported, and there was no recurrence of pain after returning to gymnastics. It's plausible that ESWT contributed favorably in terms of promoting bone formation and alleviating pain, potentially aiding in the reduction of the withdrawal period and facilitating an early return to competition.

In case 2, handstand-position full upper limb radiography was utilized for determining the treatment approach for proximal humeral epiphysiolysis accompanied by a shortened upper limb length. To date, there have been no reports on full upper limb radiography in the handstand position. When deformity or shortening of the humerus is symptomatic, surgical treatments such as osteotomy or limb-lengthening procedures may be chosen [19]. However, the patient was not aware of the shortened upper limb length. It was inferred that if the chronic shortening of the upper limb length could be unconsciously adjusted by the movements of the scapula, there would likely be no impact on the performance of gymnastics. Unlike the case 1 patient, the case 2 patient already had early closure of the growth plates, and it was considered not appropriate for ESWT, which could promote ossification. Therefore, ESWT was not performed, and only conservative treatment involving rehabilitation and careful observation was undertaken. Indeed, based on observations up to the age of 20 when growth had ceased, no particular issues arose, leading to the belief that continued monitoring was a reasonable choice.

## Conclusions

In this report, we presented the use of ESWT and handstand-position radiography in the treatment of proximal humeral epiphysiolysis. ESWT and handstand-position radiography for proximal humeral epiphysiolysis are innovative approaches that have not been previously reported. ESWT and handstand-position radiography have been suggested as potentially useful methods for treating proximal humeral epiphysiolysis in elite gymnasts. This is a case report, and further research is needed to demonstrate the efficacy of these treatments.
